# Breast Carcinoma Coexistent with Ipsilateral Axillary Non-Hodgkin Lymphoma: Cytological Diagnosis Aided with Cellblock Immunohistochemistry

**DOI:** 10.30699/IJP.2024.2014737.3203

**Published:** 2024-02-15

**Authors:** Krishnendu Mondal, Rupali Mandal

**Affiliations:** Department of Laboratory Medicine, Woodland Hospital, Shillong, Meghalaya, India

Dear Editor, 

The emergence of another primary malignancy is quite common in patients who received treatment for any previous malignancy. The mutagenic influences from chemo-radiotherapy, common genetic predisposition, and environmental factors play the causative role. But concomitant occurrence of 2 primary malignancies of different histogenesis is unusual (1). Among all cancer-related casualties, only around 0.8% of cases harbor multiple coexistent primaries. As such rarely patients suffer from synchronous non-Hodgkin lymphoma (NHL) and breast carcinoma (2). 

**Fig. 1:**
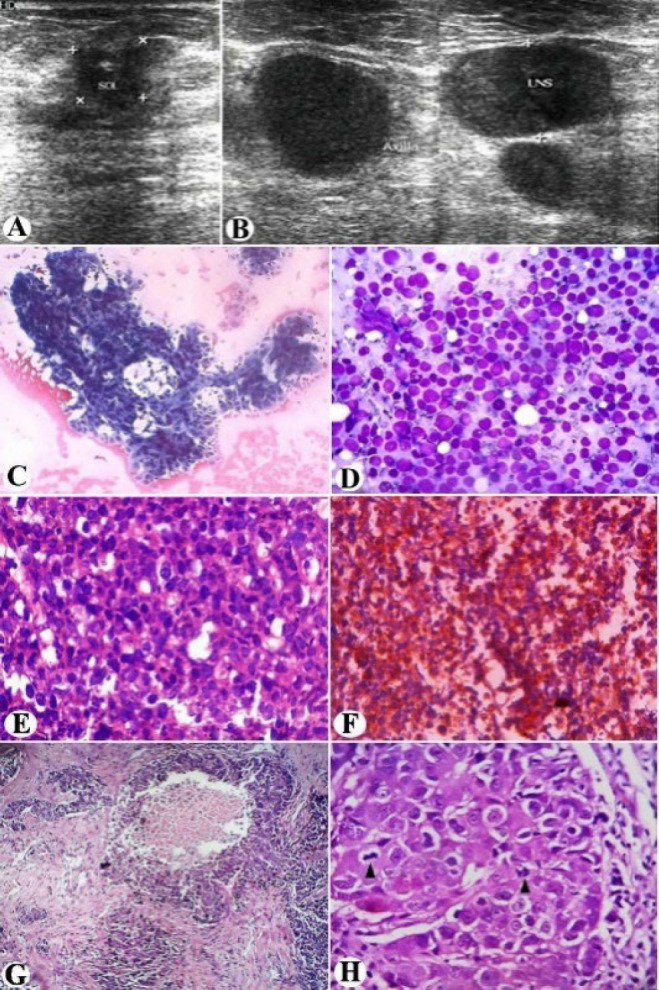
Sonographically, (A) heteroechoic breast mass having fuzzy borders and internal calcifications; Axillary lymph nodes on ultrasound: (B) Circumscribed hilum-less pseudocystic in appearance, featuring posterior acoustic shadows; FNAC from breast mass: (C) Clustered epithelial cells containing pleomorphic hyperchromatic nuclei and pale foamy cytoplasm [Pap stain, ×100]; Lymph nodes on cytology: (D) Monotonous population of large lymphoid cells reminiscent of large cell NHL [Leishman-Giemsa stain, ×400]. Cell-block (E) showing monomorphic large lymphoid cells in sheets [Haematoxylin and eosin stain, ×400]; (F) Diffuse granular cytoplasmic reactivity for CD20 by the neoplastic lymphoid cells on cell-block IHC [×100]; Histologically (G) breast mass isolated as invasive breast carcinoma exhibiting classic comedo-type necrosis [Haematoxylin and eosin stain, ×40]; On magnification (H) pleomorphic epithelial cells appear to have large vesicular nuclei, prominent nucleoli and frequent (arrow-heads) mitotic figures [Haematoxylin and eosin stain, ×400].

**Fig. 2:**
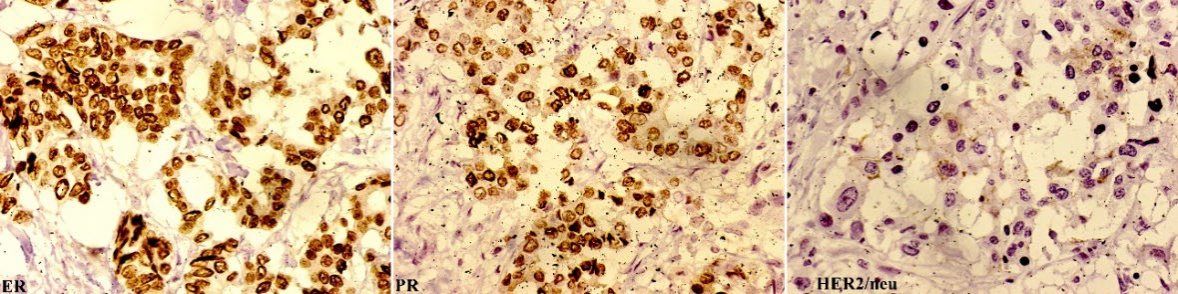
Immunohistochemically, the breast carcinoma is positive for estrogen and progesterone receptors, and negative for HER2/neu [Immunohistochemistry, ×400].

A 70-year-old patient presented with a firm mass in her right breast. Ultrasound emphasized it as a heterogeneously hypoechoic lesion having fuzzy borders and internal calcifications (Figure 1A). On simultaneous right axillary screening, multiple enlarged lymph nodes were detected in congregates. The nodes appeared sharply defined, imparting homogeneous hypoechogenicity, and posterior acoustic enhancement; but devoid of their hilum and any echogenic granulation suggestive of metastatic foci (Figure 1B). Positron emission tomography (PET/CT) did not reveal any further deposits, apart from the right breast and axillary pathology. FNAC from the breast mass expressed discohesive clusters of atypical epithelial cells, bearing hyperchromatic irregular nuclei, coarser chromatin, prominent nucleoli, and a moderate amount of pale-vacuolated cytoplasm (Figure 1C). On the contrary, aspirates from right axillary lymph nodes demonstrated monomorphic large lymphoid cells in dispersal, featuring a high nuclear-cytoplasmic ratio, rounded nuclei, condensed fine chromatin, conspicuous small nucleoli, and scanty cytoplasmic rim. Metastatic epithelial clusters were missing on the smears (Figure 1D). Given such nodal cytomorphology against the backdrop of its dubious ultrasound appearance, a suspicion of NHL was favored. Cell-block preparation with nodal aspirates resembled the FNAC. There are monotonous large lymphoid cells of identical cytomorphology arrayed in diffuse sweeps (Figure 1E). An optimal IHC panel comprising CD45, CD3, CD19, CD20, and EMA was implemented on the cell block. The lymphoid cells stained positively with leukocyte common antigen CD45 also expressed moderate reactivity with CD19. IHC with CD20 antigen displayed diffuse strong granular cytoplasmic reactivity within the lymphoid cells advocating their monoclonal B-cell origin (Figure 1F). CD3 and EMA were negative. Thereby the axillary lymphadenopathy was diagnosed as primary diffuse large B-cell lymphoma (DLBCL). Sentinel lymph node (SLN) status emerged negative for metastases but manifested lymphomatous infiltration. The patient was readily ascribed to combination chemotherapy aimed towards the NHL. After the six cycles, a modified radical mastectomy was undertaken. On complete right axillary dissection, three nodes exhibited residual lymphoma. However, any metastatic invasion by the breast carcinoma could not be confirmed on serial sections. The breast mass was confirmed as invasive breast carcinoma of no special type (IBC-NST) (Figure 1G-H), stage pT1N0M0. Estrogen receptor (ER) and progesterone receptor (PR) were positive; HER2/neu was negative (Figure 2). Hormonal therapy was reserved for cancer management. Repeat PET/CT ruled out any further dissemination confirming the DLBCL as stage I.

Synchronous malignancies are defined as the occurrence of two primaries within 6 months. While researching PET/CT-guided staging of NHLs, Papajík *et al.* (3) encountered synchronous non-lymphoid malignancy in 2.9% of their cases. Recent review literature aimed at exploring the diversity of solid tumors occurring in synchronicity with lymphomas, deduced breast cancer in only 7.7% of these cases (4). Patients treated for Hodgkin lymphoma are more susceptible to developing breast carcinoma than any other cancer. But they seldom appear synchronously. Synchronous malignancies reported alongside breast cancer include NHL, acute myeloid leukemia, pheochromocytoma, and melanoma. Therein the NHL subtypes are small lymphocytic lymphoma, mantle cell lymphoma, mucosa-associated lymphoid tissue (MALT) lymphoma, follicular lymphoma, DLBCL, and Waldenström-subtype lymphoma. Still, a concurrent axillary NHL with primary breast carcinoma has been unusual (1,2).

Like the present case, multihistogenic synchronous primaries may arise from immunological disarray triggered by the NHL, common genetic predisposition, viral etiopathogenesis as evidenced in mice models, or can be idiopathic. However, a consensus is yet to be reached on any of these phenomena (5).

The lymphoma cells often obliterate the afferent lymphatic channels en route to affected lymph nodes. These cells may also intercept the tumor necrosis factor and interleukin-mediated endothelial adhesion of breast carcinoma cells. This pathognomy likely explains the absence of axillary metastasis in concurrent breast carcinoma as in the current report (6).

Coexistent primary malignancies pose problematic to diagnose, especially when the pathology is limited within an organ and its draining lymphatic site. Individual guidelines on their staging and therapy are often little useful (7,8). In the currently reported case, the patient presented with a breast mass with ipsilateral axillary lymphadenopathy. Metastatic breast carcinoma was easily suspected. On ultrasonography, the lymph nodes appeared uniformly hypoechoic with posterior acoustic shadows resembling a cyst. Internal calcification or spicules pertinent to metastases were absent. However radiological features between metastatic and lymphomatous nodes frequently overlap. For its discrimination, most researchers successfully utilized histopathology and IHC as supportive. In a similar context, Hiraoka *et al*. (9) bumped into an unprecedented diagnosis of follicular lymphoma in the sentinel lymph node of a cT1cN0M0 stage I invasive ductal carcinoma. A comprehensive exercise of IHC was applied to isolate the definitive diagnosis. Previously Siddiqui *et al*. (10) cytologically diagnosed a collision tumor of primary ductal carcinoma and NHL. For confirmation, they still required histopathological corroboration. On the contrary in the discussed case, FNAC was followed by cell block preparation, and CD20 IHC provided the definite diagnosis. Chemotherapy was sharply commenced for the DLBCL. Specific therapy for early-stage breast cancer was deferred.

The clinicians also face trouble managing synchronous malignancies, like the one described here. When the SLN study emerges negative, the rationality of complete axillary dissection becomes questionable though it is required for staging of breast carcinoma. Also, a decision has to be prioritized regarding which malignancy should be treated first (2). While managing a similar patient, Anavekar *et al.* (11) refrained from complete axillary dissection after a negative SLN assay. But about 7.3-8.4% of cases of SLN biopsy come out false negative. So many researchers prefer to perform complete axillary dissection irrespective of the SLN status (5). SLN in the discussed patient was free from any metastasis. But considering the aggressiveness of DLBCL, which was cytologically diagnosed in advance, definitive therapy for the same was instituted ahead of the stressful modified radical mastectomy. Interval axillary dissection though did not reveal any metastasis from the breast cancer.

This latest study concludes that enlarged regional lymph nodes in any carcinoma may not always indicate metastatic invasion. Early cytological diagnosis of an aggressive second malignancy like the NHL provides prognostic advantages. Implicit multifaceted review on a case-to-case basis is needed to chalk out the optimal treatment protocol for these patients.
